# Clinical factors associated with a *Candida albicans *Germ Tube Antibody positive test in Intensive Care Unit patients

**DOI:** 10.1186/1471-2334-11-60

**Published:** 2011-03-09

**Authors:** Javier Pemán, Rafael Zaragoza, Guillermo Quindós, Miriam Alkorta, María S Cuétara, Juan J Camarena, Paula Ramírez, María J Giménez, Estrella Martín-Mazuelos, María J Linares-Sicilia, José Pontón

**Affiliations:** 1 Department of Microbiology, Hospital Universitario La Fe, (Av Campanar, 21), Valencia, (46009), Spain; 2 Intensive Care Unit, Hospital Universitario Dr. Peset, (Gaspar Aguilar, 90), Valencia, (46017), Spain; 3 Departament of Immunology, Microbiology and Parasitology, Facultad de Medicina y Odontología, Universidad del País Vasco, (Apdo 699), Bilbao, (48080), Spain; 4 Department of Microbiology, Hospital de Zumarraga, (Argixao Taldea s/n), Zumarraga, (20700), Spain; 5 Department of Microbiology Hospital Severo Ochoa, (AV Orellana s/n), Leganés, (28911), Spain; 6 Department of Microbiology, Hospital Universitario Dr. Peset, (Gaspar Aguilar, 90), Valencia, (46017), Spain; 7 Intensive Care Unit, Hospital Universitario La Fe, (Av Campanar, 21), Valencia, (46009), Spain; 8 Department of Microbiology Hospital N.S. de Valme, (Ctra. Cádiz, km 548), Seville, (41014), Spain; 9 Department of Microbiology, Facultad de Medicina, (Av Menéndez Pidal s/n), Córdoba, (14004), Spain

## Abstract

**Background:**

Poor outcomes of invasive candidiasis (IC) are associated with the difficulty in establishing the microbiological diagnosis at an early stage. New scores and laboratory tests have been developed in order to make an early therapeutic intervention in an attempt to reduce the high mortality associated with invasive fungal infections. Candida albicans IFA IgG has been recently commercialized for germ tube antibody detection (CAGTA). This test provides a rapid and simple diagnosis of IC (84.4% sensitivity and 94.7% specificity). The aim of this study is to identify the patients who could be benefited by the use of CAGTA test in critical care setting.

**Methods:**

A prospective, cohort, observational multicentre study was carried out in six medical/surgical Intensive care units (ICU) of tertiary-care Spanish hospitals. *Candida albicans *Germ Tube Antibody test was performed twice a week if predetermined risk factors were present, and serologically demonstrated candidiasis was considered if the testing serum dilution was ≥ 1:160 in at least one sample and no other microbiological evidence of invasive candidiasis was found.

**Results:**

Fifty-three critically ill non-neutropenic patients (37.7% post surgery) were included. Twenty-two patients (41.5%) had CAGTA-positive results, none of them with positive blood culture for *Candida*. Neither corrected colonization index nor antifungal treatment had influence on CAGTA results. This finding could corroborate that the CAGTA may be an important biomarker to distinguish between colonization and infection in these patients. The presence of acute renal failure at the beginning of the study was more frequent in CAGTA-negative patients. Previous surgery was statistically more frequent in CAGTA-positive patients.

**Conclusions:**

This study identified previous surgery as the principal clinical factor associated with CAGTA-positive results and emphasises the utility of this promising technique, which was not influenced by high *Candida *colonization or antifungal treatment. Our results suggest that detection of CAGTA may be important for the diagnosis of invasive candidiasis in surgical patients admitted in ICU.

## Background

Invasive candidiasis (IC), especially in the critical care setting, has become an excellent target for prophylactic, empiric, and pre-emptive therapy interventions due to its increased incidence, high morbidity, mortality rate, and associated healthcare costs [1,2]. Although the past few years have brought exciting developments in antifungal pharmacotherapy, the mortality rate remains excessively high [3-7]. Poor outcomes are associated with the difficulty in establishing the microbiological diagnosis at an early stage of IC (blood culture results are positive in only 50% and the antigen or antibody detection also show low sensitivity rates at the present time) [2] and the delay in receiving antifungal treatment [8,9]. Moreover, it is well known that inadequate empirical antibiotic treatment is an independent determinant of hospital mortality and mycoses are among the types of infection with the highest rates of inappropriate initial treatment [10] and consequently worse prognosis [11].

New scores and laboratory tests have been developed in order to make an early therapeutic intervention in an attempt to reduce the high mortality associated with invasive fungal infections [12,13]. Substantial progress has been made in diagnosis of IC with the development of a variety of methods for the detection of antibodies and antigens. However, no single test has found widespread clinical acceptance and there is a consensus that diagnosis based on a single specimen lacks sensitivity [14,15].

Candida albicans IFA IgG has been recently commercialized for *Candida albicans *germ tube antibody (CAGTA) detection. This test is based on the detection, by an indirect immunofluorescence assay, of antibodies against the surface of *C. albicans *germ tubes. The test provides a rapid and simple diagnosis of IC in the clinical microbiology laboratory (84.4% sensitivity and 94.7% specificity) [16,17]. Although the performance of the test has been studied in haematological patients (87.5% sensitivity and 95.2% specificity) [18], no large clinical study has been performed in the intensive care unit (ICU) setting with this new tool. Two recently published mortality analyses of our group showed a significant decrease of mortality in those patients with a CAGTA-positive result in ICU patients [19,20], especially in those with patterns of increasing CAGTA titres who had been treated with antifungal agents. Thus, antifungal treatment should be considered when CAGTA titres increase in critically ill patients [20]. However, the limitation of these data, the complexity and the cost of this technique make it mandatory to find which patients could be benefit by the determination of CAGTA in order not to apply this technique universally. For these reasons, the aims of the present study were to determine the clinical features of the patients with a CAGTA-positive result in the ICU setting, in an attempt to define the population of critically ill patients who could be benefit by this technique analyzing the factors associated to a positive result in order to make rentable the use of this test.

## Methods

### Patients and methods

During a two-year period (January 2005-December 2006) a prospective observational multicenter study was conducted at six Spanish University hospitals: Hospital Doctor Peset -HDP- (563 hospital beds/16 ICU beds, Valencia), Hospital La Fe -HLF- (1,292/21, Valencia), Hospital de Cruces -HDC- (931/18, Barakaldo), Hospital Severo Ochoa -HSO- (365/12, Leganés), Hospital Reina Sofía -HRS- (1,301/34, Córdoba), and Hospital Nuestra Señora de Valme -HNSV- (639/14, Seville). The cohort described is the same as has been previously published by our group [19,20], but the data showed here are novel and have not been included in our cited articles. The institutional review boards at each participating institution approved the study and informed consent was obtained in each patient included in the study.

### Study design and data collection

The study was developed following a pre-designed protocol with several predetermined analyses. A critically ill patient was eligible for investigation and was enrolled in the study if any of the following five inclusion criteria were present: i) acute pancreatitis of more than seven days of evolution; ii) prolonged ICU stay (>14 days) and three or more risk factors (diabetes mellitus, extra-renal depuration, parenteral nutrition, more than seven days of broad spectrum antibiotic therapy, and major abdominal surgery); iii) liver transplant; iv) neutropenia or bone marrow transplant (BMT); and v) high *Candida *colonization. Exclusion criteria were as follow: i) pregnancy; ii) younger than 18 years old, iii) previous IC; or iv) life expectancy lower than seven days. Prior antifungal therapy was not an exclusion criterion. All patients were consecutively included in the study.

Severity of illness at inclusion of study was calculated with the APACHE II score (Acute Physiology and Chronic Health Evaluation). For all eligible patients, screening cultures for *Candida *colonization were performed weekly until seven days after ICU discharge. These samples were obtained from bronchial aspirates, oropharynx, urine, rectum, and peri-catheter skin as part of the surveillance study. A corrected colonization index (CCI) was used to assess the intensity of *Candida *colonization [21-23], and patients with CCI ≥0.4 were considered as highly colonized. J. Pemán and R. Zaragoza reviewed all patients' forms before including them in the database.

### Microbiological studies

CAGTA detection (*Candida albicans *IFA IgG, Vircell, Spain) was performed twice a week. A positive result, defined as serologically proven candidiasis, was considered when a serum titre ≥1:160 was detected in at least one sample and no other microbiological evidence of IC was found. Blood and sterile sites cultures were drawn following physicians' criteria and processed with automated systems (BACTEC, Becton Dickinson Diagnostic Instrument Systems, Madrid, Spain or BacTAlert, bioMérieux España, Madrid, Spain). Identification of yeasts at species level was made with the API 20C, API 32C, or the YST card of the Vitek system (bioMérieux España, Madrid, Spain) at each institution. The decision to add antifungal therapy for patients with suspected IC was at the discretion of the prescribing physician based on clinical criteria, but it was not influenced by CAGTA results (physicians had no access to the results). Clinical, microbiological and outcome variables were recorded.

### Data analysis

All quantitative data are presented as mean ± SD unless otherwise stated. The chi-square or Fisher's exact test was used to compare categorical variables and student's t-test was applied for quantitative homocedastic variables with normal distribution. Two tailed *p *value of 0.05 or less was considered for statistical significance. Data analysis was performed with the SPSS software for Windows version 11.5 (SPSS, Chicago, IL). An external statistical company assessed this analysis independently.

## Results

Fifty-three miscellaneous critically ill patients were included in the study, 37.7% of whom needed surgical procedure during their stay in the ICU. The hospital distribution of patients was as follows: 17 (HDC), 12 (HLF), 11 (HDP), 9 (HSO), 3 (HNSV) and 1 (HRS). The most frequent patient inclusion criteria were prolonged ICU stay and presence of ≥3 IC risk factors (60%) followed by CCI ≥0.4 (23%), neutropenia or BMT (7.5%), acute pancreatitis (6%) and liver transplant (4%). Five patients (9%) presented extended ICU stay and ≥3 IC risk factors plus high CCI. The age and APACHE II score averages were 71.5 ± 18.4 years, and 14.9 ± 5.4 points, respectively. The principal causes of ICU admission were septic shock (28.3%) and respiratory failure (28.3%), followed by post-surgery standard care (24.5%) and coma (9.4%). The patient groups did not differ significantly in demographic characteristics or inclusion criteria among participating centres.

Culture-based methods yielded negative for blood and sterile sites cultures for *Candida *spp. when where drawn in all study patients. However, 43.3% of the patients were highly colonized during the study.

Twenty-two patients (41.5%) had CAGTA-positive results (10 patients had one positive sample, 8 had two, and 4 had ≥3 positive determinations), none of them with positive blood culture for *Candida*. The CAGTA titres observed in these patients ranged from 1/160 to 1/2560. There were no differences between CAGTA-positive and -negative patients in age, sex, inclusion criteria, cause of admission, and presence of hepatic failure. The presence of acute renal failure at the beginning of the study was more frequent in CAGTA-negative patients, but no extra-renal depuration was needed. APACHE II score was also statistically higher in this group. Previous surgery was statistically more frequent in CAGTA-positive patients in the univariate analysis (Table 1). No statistical differences were found, in the rate of highly colonized patients, between positive and negative CAGTA results at the end of the study (77.2% vs. 89.6%, respectively; *p *= 0.26).

**Table 1:** Main clinical characteristics of patients according to CAGTA results

	**No. of patients (%)**			
		
	**Total**	**CAGTA +**	**CAGTA -**	***p *value**
	
Patients	53	22 (41.5)	31 (58.5)	-
Age (years)	71.5 ± 18.4	69.2 ± 22.2	73.1 ± 15.3	0.46
Male:Female (ratio)	1.65	1.2	2.3	0.25
APACHE II (score)	14.9 ± 5.4	12.2 ± 4.4	16.9 ± 5.2	0.001
Septic shock	15 (28.3)	4 (18.2)	11 (35.4)	0.16
Respiratory failure	15 (28.3)	7 (31.8)	8 (25.8)	0.63
Coma	5 (9.4)	2 (9.1)	3 (9.7)	0.92
Renal failure	24 (45.3)	6 (27.3)	18 (58.1)	0.02
Extra-renal depuration	16 (30.2)	4 (18.2)	12 (38.7)	0.1
Hepatic failure	19 (35.8)	9 (40.9)	8 (25.8)	0.24
Neutropenia	4 (7.5)	0	4 (12.9)	0.1
Broad spectrum antibiotics	36 (67.9)	17 (77.3)	19 (61.3)	0.21
Parenteral nutrition	33 (62.3)	14 (63.7)	19 (61.3)	0.86
Hepatic transplant	2 (3.8)	1 (4.5)	1 (3.2)	0.80
Acute pancreatitis	3 (5.7)	2 (9.1)	1 (3.2)	0.36
Diabetes mellitus	12 (22.6)	4 (18.2)	8 (25.8)	0.51
Previous surgery	20 (37.7)	12 (54.5)	8 (25.8)	0.03

CAGTA: *Candida albicans *germ tube antibody

Empiric antifungal treatment was applied in 50.9% of cases during the study (after the inclusion); the regimens most frequently prescribed were fluconazole (48.1%) and voriconazole (14.8%). There was no difference in the global administration of antifungal treatment between CAGTA-positive and -negative patients (45.4% vs. 54.8%; p = 0.45); however, the use of voriconazole showed a tendency to be more frequent in CAGTA-positive patients (5.9% vs. 30%; *p *= 0.08) (Table 2).

**Table 2:** Distribution of antifungal treatment administered according to CAGTA results.

	**No. of patients (%)**			
		
	**Total**	**CAGTA +**	**CAGTA -**	***p *value**
	
Fluconazole	13 (48.1)	6 (60)	7 (41.2)	0.34
Voriconazole	4 (14.8)	3 (30)	1 (5.9)	0.08
Fluconazole + caspofungin*	4 (14.8)	0	4 (23.5)	0.09
Liposomal amphotericin B	2 (7.4)	0	2 (11.8)	0.25
Liposomal amphotericin B + fluconazole*	2 (7.4)	0	2 (11.8)	0.25
Caspofungin	1 (3.7)	0	1 (5.8)	0.43
Fluconazole + liposomal amphotericin B +caspofungin*	1 (3.7)	1 (10)	0	0.18
				
Total	27 (100)	10 (100)	17 (100)	-

CAGTA: *Candida albicans *germ tube antibody

*Sequential treatment

## Discussion

Mortality rates of IC episodes in the critical care setting remain excessively elevated due to diagnosis difficulties and, subsequently, inappropriate treatment. IC diagnosis usually requires a high index of suspicion and is difficult because the infection lacks pathognomonic signs, blood cultures are often negative and, in many instances, it is not possible to obtain specimens for histology; therefore, serology could be an aid in its diagnosis, as it provides specific biomarkers [2,24,25]. Demonstration of IC may not be apparent because the infectious burden is beneath the analytical sensitivity of the chosen diagnostic modality, or infection remains limited in the context of neutrophil recovery or follows administration of prophylactic or empirical antifungal therapy. Serological tests for the diagnosis of IC by detecting antibodies against different antigens of *Candida *must differentiate *Candida *colonization or superficial infection from tissue invasion and candidemia requiring antifungal therapy. Sera from patients with IC recognize a mannoprotein of 230-250 kDa located on the germ tube cell wall surface. Pontón et al. [26,27] have developed an indirect immunofluorescence assay to detect antibodies (CAGTA) against this antigen present in *C. albicans *germ tubes, which has been a useful biomarker for the diagnosis of IC in different groups of patients, including intravenous heroin users, BMT recipients, patients with haematological disorders and ICU patients [16,18,28,29]. The test has shown an overall sensitivity of 77-89% and a specificity of 91-100%. Although the titres found in immunocompromised patients are lower than those found in immunocompetent patients, the overall performance of the test is similar. Detection of CAGTA in patients with invasive infections caused by *Candida *species other than *C. albicans *(*Candida tropicalis*, *Candida parapsilosis*, *Candida glabrata*, *Candida dubliniensis*, *Candida guilliermondii *and *Candida krusei*) may also be positive, although titers are lower than in candidiasis by *C. albicans *[16,27,29-31].

*Candida albicans *IFA IgG has been recently commercialized for CAGTA detection. This test has been compared in a retrospective study to the standard test using 172 sera from 51 haematological and intensive care patients [16]. The commercially available test was similar to the standard test and provided faster and easier diagnosis of IC in the clinical microbiology laboratory.

To our knowledge, this is the first time that the new commercial kit for CAGTA detection has been studied in the ICU setting. It is well known that the global sensibility rate of blood culture is not too high (50%) in the hospital population [32]. Moreover, this ratio is considerable lower in the ICU setting as León et al. [12] observed in the *Candida *score study, where the ratio of proven IC was only 5.7%. However, until now this procedure is still the gold standard for candidemia diagnosis. The reasons for the low number of positive blood cultures in critical care setting are unknown but are likely to be related to the prophylactic and empiric treatments administered to these patients [33,34]. In our study, no blood cultures were positive. The low ratio of positive blood cultures in critically ill patients obtained in all studies in this setting, including this one, confirms the compelling necessity of finding another sound gold standard for validating new diagnostic tools [12,35,36]. Then, serological data may be used as evidence to implicate *Candida *spp. when mycological and/or histological data are negative or cannot be acquired. The high prevalence of CAGTA-positive results in the population studied (41.5%) corroborates the adequacy of the inclusion criteria used in this study as a predictive biomarker of *Candida *infection and the need of consolidating the CAGTA determination in a delimited group of ICU population. For these reasons and for the excellent sensitivity and specificity previously reported [16], all the CAGTA-positive patients could be considered as "probable" IC. The preliminary results presented in this study evidence the need for a large multicentre study to validate the CAGTA detection as an alternative diagnosis tool in the ICU setting [16,30].

This study identifies the principal risk factors associated with serological proven candidiasis (Table 1) and, probably, with hidden invasive *Candida *infection in the ICU population due to the absence of other microbiological positive tests. Only previous surgery was statistically more frequent in patients with CAGTA-positive results. Furthermore, in our study previous surgery is a determinant risk factor for candidemia as has been described by León et al. [12]; however, although diabetes mellitus has been previously described as a risk factor for developing IC in critically ill patients [37], our findings do not support the inclusion of diabetes mellitus in clinical scores to predict IC in ICU patients. Recently published clinical scores such as the "Candida score" [12] or the Ostrosky-Zeichner et al. rule [38] have not included this risk factor. Consequently, periodical CAGTA determination should be mandatory especially in ICU surgical patients to achieve an early diagnosis and, therefore, to improve the candidemia prognosis, especially in those patients with patterns of increasing CAGTA titres [20]. This assay could improve the predictor value of these previously described prediction scores and rules.

The rate of highly colonized patients was very high as has been previously described [39] and no statistical differences were found between CAGTA-positive and -negative patients at the end of the study. This finding could corroborate the CAGTA technique as a key biomarker to distinguish between colonization and infection in these patients.

In our study, the decision to add empiric antifungal therapy for patients with suspected IC was at the discretion of the prescribing physician based on clinical criteria, but was never guided by CAGTA results. The no difference observed neither the global administration of antifungal treatment nor antifungal agent used between CAGTA-positive and -negative give more value to this technique avoiding false-negative results in presence of any antifungal therapy.

Several limitations must be noted in this study. First, the small number of patients due to the difficulty to enrol this kind of patients with predefined criteria. Moreover, there was no possibility to establish the sensibility and specificity of CAGTA technique due to the absence of microbiological and histological documentation of IC in this study.

## Conclusions

This study identified previous surgery as the principal clinical factor associated with CAGTA-positive results (serologically proven candidiasis) and emphasises the utility of this promising technique, which was not influenced by high *Candida *colonization or antifungal treatment. For these reasons the addition of the CAGTA technique to the armamentarium for the diagnosis of IC in surgical patients admitted in ICU would be a great advantage as it could help to identify hidden invasive *Candida *infection in the surgical ICU population with a long stay in this setting.

## Competing interests

The authors declare that they have no competing interests.

## Authors' contributions

JaP, RZ, GQ and JoP designed the study. RZ, PR and EMM collected the data; MA, MSC, JJC, MJG, EMM, MJL performed the technique. JaP, RZ made the interpretation of statistical analyses. JaP. RZ and GQ wrote the paper with input from all the authors who each approved the final version.

## Pre-publication history

The pre-publication history for this paper can be accessed here:

http://www.biomedcentral.com/1471-2334/11/60/prepub
